# Cardiovascular risk and encoding-related hippocampal connectivity in older adults

**DOI:** 10.1186/s12868-019-0518-4

**Published:** 2019-07-31

**Authors:** Liesel-Ann C. Meusel, Carol E. Greenwood, Andrea Maione, Ekaterina Tchistiakova, Bradley J. MacIntosh, Nicole D. Anderson

**Affiliations:** 1 Rotman Research Institute, Baycrest, 3560 Bathurst Street, Toronto, ON M6A 2E1 Canada; 2 Department of Nutritional Sciences, Faculty of Medicine, University of Toronto, Toronto, ON Canada; 3 Heart and Stroke Foundation Canadian Partnership for Stroke Recovery, Sunnybrook Research Institute, Toronto, ON Canada; 4 Department of Medical Biophysics, Faculty of Medicine, University of Toronto, Toronto, ON Canada; 5 Departments of Psychology and Psychiatry, University of Toronto, Toronto, ON Canada

**Keywords:** Older adults, Cardiovascular risk, fMRI, Connectivity, Memory, Encoding

## Abstract

**Background:**

Cardiovascular conditions contribute to brain volume loss, reduced cerebrovascular health, and increased dementia risk in aging adults. Altered hippocampal connectivity has also been observed in individuals with cardiovascular conditions, yet the functional consequences of these changes remain unclear. In the present study, we collected functional magnetic resonance imaging data during memory encoding and used a psychophysiological interaction analysis to examine whether cardiovascular burden, indexed using the Framingham risk score, was associated with encoding-related hippocampal connectivity and task performance in cognitively-intact older adults between 65 and 85 years of age. Our goal was to better understand the early functional consequences of vascular and metabolic dysfunction in those at risk for cognitive decline.

**Results:**

High Framingham risk scores were associated with lower total brain volumes. In addition, those with high Framingham risk scores showed an altered relationship between left hippocampal-medial prefrontal coupling and task performance compared to those with low Framingham risk scores. Specifically, we found a significant interaction of Framingham risk and learning on connectivity between the left hippocampus and primarily left midline prefrontal regions comprising the left ventral anterior cingulate cortex and medial prefrontal cortex. Those with lower Framingham risk scores showed a pattern of weaker connectivity between left hippocampal and medial prefrontal regions associated with better task performance. Those with higher Framingham risk scores showed the opposite pattern; stronger connectivity was associated with better performance.

**Conclusions:**

Findings from the current study show that amongst older adults with cardiovascular conditions, higher Framingham risk is associated with lower brain volume and altered left hippocampal-medial prefrontal coupling during task performance compared to those with lower Framingham risk scores. This may reflect a compensatory mechanism in support of memory function and suggests that older adults with elevated cardiovascular risk are vulnerable to early Alzheimer disease-like dysfunction within the episodic memory system.

## Background

Cardiovascular conditions (hypertension, type 2 diabetes, high cholesterol) in middle-aged and older adults contribute to structural brain changes and volume loss, reduced cerebrovascular health, subtle cognitive deficits, and increased dementia risk (e.g., [1–4]). Metabolic dysregulation in particular is detrimental to the integrity of the hippocampus and associated medial temporal lobe regions (e.g., [5–7]), and is implicated in Alzheimer disease pathology [8, 9]. Consistent with this, studies have found that middle-aged and older adults with type 2 diabetes show Alzheimer-like memory difficulties and medial temporal lobe atrophy (e.g., [5, 10]), and other work has shown a higher incidence of amnestic mild cognitive impairment, the prodrome to Alzheimer disease, in older adults with familial hypercholesterolemia [11].

Changes in hippocampal connectivity and default mode network integrity have also been found in individuals with cardiovascular conditions, changes that parallel those seen in mild cognitive impairment and Alzheimer disease. In one study, relative to healthy subjects, older adults with type 2 diabetes showed a pattern of reduced resting-state connectivity between the hippocampus and key default mode regions [12], and another found insulin resistance in middle-aged women to be associated with reduced resting-state connectivity between the hippocampus and medial prefrontal cortex [13]. The functional consequences of these changes remain unclear, however; while both studies found evidence of cognitive deficits in their patient group (i.e., poorer memory and executive function on neuropsychological testing, relative to healthy controls), neither looked at the direct associations between test performance and connectivity. Furthermore, to our knowledge there are no studies of adults with cardiovascular conditions that have looked at functional connectivity during a cognitive task (i.e., rather than rest).

In the Alzheimer disease literature, altered functional integrity of the hippocampus and the default mode network has emerged as an indicator of incipient pathology, often before brain volume loss or behavioral changes are evident [14, 15]. It has been suggested that normal, age-related changes in hippocampal and hippocampal-cortical connectivity result in a functionally isolated hippocampal complex at rest, reduced hippocampal-cortical connectivity during encoding, and reduced performance on episodic memory tasks [16]. There appears to be an initial period of hyperactivity and hyperconnectivity in central executive and medial temporal networks primarily (e.g., [17]); however, reduced connectivity is the more dominant pattern reported in mild cognitive impairment and Alzheimer disease, starting in default mode regions and moving with disease progression into posterior, medial temporal, and then frontal networks [18]. Reduced connectivity is thought to be a result of increasing Alzheimer disease pathology, and early disconnection within the default mode network relative to other brain networks may reflect the susceptibility of these regions to this process [19].

The most common explanation for hyperconnectivity is compensation (see [18] for discussion), interpreted as increased neural recruitment to meet task demand in the face of network disruption. Positive associations between connectivity metrics and behavioral performance offer some evidence for hyperconnectivity as a compensatory process. For example, better memory task performance is associated with stronger resting-state connectivity (i.e., hyperconnectivity) between the hippocampus and posteromedial cortex in cognitively-intact older adults [20], and, similarly, better task switching performance is associated with stronger dorsolateral prefrontal-occipitotemporal connectivity [21]. Some studies show the opposite pattern, however, across both task and rest (i.e., stronger connectivity associated with reduced performance; [22, 23]). While this may reflect “attempted compensation” (i.e., unsuccessful attempts at compensation; [24]), there are other issues to consider. For instance, hyperconnectivity can have competing effects on task performance, and more generally carries the cost of reduced efficiency and cognitive fatigue [18].

The objective of the present study was to determine whether cardiovascular burden in cognitively-intact older adults is associated with early functional brain changes (i.e., before cognitive deficits emerge). We used a psychophysiological interaction (PPI) analysis to examine functional connectivity between the hippocampus and the rest of brain during memory encoding, which allows for the measurement of task-specific changes in the interactions between two brain regions. This analysis identifies those brain areas where activity is more strongly associated with activity in a seed region of interest, during a particular cognitive operation (i.e., stimulus encoding), and after accounting for more general, task-nonspecific connectivity between the two regions [25]. Our goal was to examine the effect of cardiovascular burden on hippocampal connectivity and task performance, to better understand the functional and behavioral consequences of vascular and metabolic dysfunction in those at risk for cognitive decline.

## Methods

### Participants

Thirty, cognitively-intact right-handed adults between 65 and 85 years of age and with at least one cardiovascular condition were recruited using an internal participant database and advertisements. The study was approved by the Research Ethics Board at Baycrest and all participants provided their written informed consent to participate.

### Inclusion/exclusion criteria

Demographic, medical, and cognitive (Modified Telephone Interview for Cognitive Status; [26]) screening questionnaires were completed over the telephone. Hypertension criteria were: ≥ 2 year self-reported history of hypertension and ≥ 2 year history of treatment with antihypertensive medications (i.e., angiotensin converting enzyme inhibitors, angiotensin II receptor blockers, or diuretics). Those with type 2 diabetes were also included in the study (≥ 2 year self-reported history of diabetes treated with diet alone or with hypoglycemic medication), provided they had no major diabetic complications (e.g., retinopathy, nephropathy and neuropathy) and did not require insulin therapy. Exclusion criteria included the presence of any other significant medical, neurological, or psychiatric disorder such as: dementia, multiple sclerosis, stroke, epilepsy, major depressive disorder, coronary heart disease, heart failure, chronic lung disease, hepatic disease, inflammatory and rheumatological disorders, inflammatory bowel disease, uncontrolled hyper- or hypothyroidism, radiation to the head, chemotherapy, and/or loss of consciousness > 5 min. Those taking centrally active medications (e.g., antidepressants, sleep medications), on hormone replacement therapy, with magnetic resonance imaging-incompatible medical devices, and/or not fluent in English by 5 years of age were also excluded. Based on a comprehensive neuropsychological assessment of estimated intelligence (vocabulary and reasoning), attention and speed of processing, memory, executive function, and mood, individuals who met criteria for mild cognitive impairment, defined as a relative weakness (> 1.5 SD below estimated-IQ) on two or more tests within the same domain, were also excluded from participation.

### Health assessment

In session 1, participants provided a fasting blood sample to measure glucose, total cholesterol, high-density lipoprotein cholesterol (HDL-C), and low-density lipoprotein cholesterol (LDL-C). All blood analyses were carried out at Mt. Sinai Hospital, Toronto, ON. We also measured weight, waist circumference, and blood pressure (BpTRU Medical Devices), taken after participants had been sitting quietly for 5–10 min. From these data, cardiovascular burden was indexed using the Framingham risk score (FRS), calculated directly from the Cox model formula [27], which considers sex, age, total cholesterol and HDL-C, systolic blood pressure, hypertension treatment status, smoking status, and diabetes status.

### Functional imaging tasks

In session 2, blood oxygenation level dependent (BOLD) functional magnetic resonance imaging (fMRI) was acquired during five separate runs of a word-list learning task, and during a breath-hold task. Because the BOLD signal is sensitive to cerebrovascular dysfunction that can accompany metabolic and vascular conditions [28, 29], the breath-hold task was used to provide an index of cerebrovascular reactivity. All tasks were administered using E-Prime 1.2 software (Psychology Software Tools, Pittsburgh, PA). The word-list learning task comprised of 16 target words presented in the same order over 5 runs (140 s; 70 TRs/run). Each item was presented for 2 s, followed by a 2- 4- or 6-s variable inter-stimulus interval. After each run, subjects were asked to recall as many words as possible (unscanned). The breath-hold task was presented over 1 run (312 s; 156 TRs) and consisted of the following sequence repeated 6 times: 2-s expiration, 15-s breath-hold, 30-s of normal breathing.

### Imaging parameters

Images were acquired on a 3 Tesla Magnetom Trio Siemens scanner with a 12-channel head coil. High-resolution structural images (T1-weighted three-dimensional magnetization-prepared rapid gradient-echo sequence) were acquired with the following parameters: TR/TE = 2000/2.63 ms, field of view = 256 mm, slice thickness = 1 mm, number of slices = 160. T2*-weighted images were collected during the study phase of the word-list learning task and the breath-hold task (TR/TE = 2000/30 ms, flip angle = 70°, field of view = 200 mm, slice thickness = 5 mm, number of slices = 32). Fluid attenuation inversion recovery (FLAIR) images (TR/TE/TI = 9000/96/2500 ms, field of view = 224 mm, slice thickness = 5 mm, number of slices = 32) were collected to assess white matter hyperintensity burden.

### Image preprocessing

Left and right hippocampi were manually traced on the native-space anatomical images in Analyze (version 7.5; Mayo Clinic, Biomedical Imaging Resource, AnalyzeDirect, Overland Park, KS, USA) by a trained research assistant according to established protocols [30, 31]. To increase the accuracy of the tracings, every slice was traced rather than every second slice. The tracings were carried out on the sagittal plane and included the hippocampus proper, subiculum, fimbria, alveus, and the dentate gyrus.

Cortical reconstruction and volumetric segmentation on the native-space anatomical images was performed with the Freesurfer image analysis suite (version 5.3.0; http://surfer.nmr.mgh.harvard.edu/), to obtain whole brain and regional brain volumes. We extracted total gray matter volume and total intracranial volume for each participant, and calculated a normalized total gray matter score by dividing total gray matter volume by total intracranial volume.

BOLD data preprocessing of the word-list learning task was carried out in AFNI (Analysis of Functional NeuroImages; Cox, 1996; ver 16.1.15; May 11, 2016) according to the following steps: physiological motion correction, removal of the first 5 TRs of each word-list learning run to allow for stabilization of the magnetic field, interleaved slice time correction, alignment of the anatomical and functional datasets, motion correction, and spatial smoothing (4 mm full width at half maximum). In addition, to prevent inclusion of regions susceptible to signal dropout in the analysis (e.g., ventral prefrontal and anterior/inferior temporal regions), we created a mask of voxels with valid EPI signal using 3dAutomask (AFNI) which was then used to constrain the individual-level PPI analysis described below. Preprocessing of the breath-hold task to generate a cerebrovascular reactivity map for each participant was carried out in FMRIB Software Library (FSL; http://www.fmrib.ox.ac.uk/fsl; [32]) and is described in detail elsewhere [3]. Preprocessing of the FLAIR images to generate the total volume of white matter hyperintensities for each participant was done following the procedures of Gibson et al. [33]. Following preprocessing, one participant was excluded because of a high white matter burden (14.3 cc), relative to the mean of the other subjects (1.2 cc). In addition, four participants were excluded because of excessive motion (either because of large motion spikes during scanning, or because a large number of TRs required censoring, based on the Euclidian norm of the motion derivative exceeding 0.3). One participant was also excluded because of experimenter error (incorrect task version administered). Ultimately, 24 participants were included in the analyses reported here.

### Psychophysiological interaction (PPI) analysis

At the individual-level, we conducted a generalized PPI analysis [34, 35] in AFNI to identify brain regions that showed changes in connectivity with the left or right hippocampus during stimulus encoding (i.e., encoding-related connectivity). Each participant’s manually-traced left and right hippocampi served as a seed region of interest. For each of the 5 runs, the mean time series was extracted across each seed region and detrended to obtain a left and right hippocampal seed time series regressor. From these two time series regressors, two PPI regressors were created by deconvolving each time series regressor and multiplying that with the stimulus presentation time series. The left and right hippocampal seed time series and the left and right hippocampal PPI regressors were then concatenated in time across the 5 runs (325 TRs). Separate, individual-level regression analyses were conducted in 3dDeconvolve for the left and right hippocampal seed regions. Within this analysis, volumes with signal outliers at more than 10% of brain voxels and/or a Euclidian norm of the head motion derivative exceeding 0.3 were censored and interpolated. The regression model consisted of: the stimulus presentation time series regressors, six “nuisance” regressors reflecting motion parameter estimates, the left/right seed time series regressor and the left/right PPI regressor. With this model, any variance in connectivity explained by the PPI regressor term (i.e., reflecting encoding-related connectivity) is orthogonal to any variance in connectivity accounted for by the seed time series regressor (i.e., reflecting more general, task-nonspecific connectivity).

Statistical parametric maps were transformed into Talairach space, using the normalized anatomical images for each participant as a template. This resulted in a “standard-space” statistical brain map for each participant, representing voxelwise connectivity strengths with the left (or right) hippocampus.

### Group analysis: cardiovascular burden and hippocampal connectivity

At the group level, using two analyses of covariance models (3dMVM; [36]), we looked for brain regions showing associations between left and right hippocampal PPI connectivity, cardiovascular burden (i.e., FRS), and cognitive task performance. Cognitive task performance was defined as the total number of words learned across all 5 trials of the word-list learning task (TotLrn). Left or right PPI connectivity were the dependent variables, and we controlled for hippocampal volume. We did not control for age because this is accounted for in the Framingham Risk calculation. In these models, a significant interaction between FRS and TotLrn on PPI connectivity is independent of any simple effect of FRS, reflecting brain regions where PPI connectivity is affected by cardiovascular burden and also associated with task performance. The resultant voxelwise statistical maps were thresholded at p < 0.01 and cluster-wise correction was applied using 3dClustSim in AFNI (ver 16.1.15), incorporating an estimated non-Gaussian autocorrelation function. Based on this, voxel clusters of n > 48 were considered significant at a family-wise alpha of p < 0.05.

### Group analysis: cardiovascular burden and cerebrovascular reactivity

There were five participants in the final sample who were non-compliant with the breath-hold paradigm, based on visual inspection of a respiration belt tracing. To determine the extent to which individual differences in cerebrovascular reactivity affected hippocampal PPI connectivity, we conducted a separate analysis using the subset of participants with valid breath-hold data. Two regression models were constructed, with left and right PPI connectivity as the dependent variable and cerebrovascular reactivity included as a voxel-wise covariate of interest. Using the same methods described above, the resultant voxelwise statistical maps were thresholded at p < 0.01 with a cluster size of n > 48 considered significant at a corrected level of *p *< 0.05.

## Results

### Sample characteristics

**Fig. 1:**
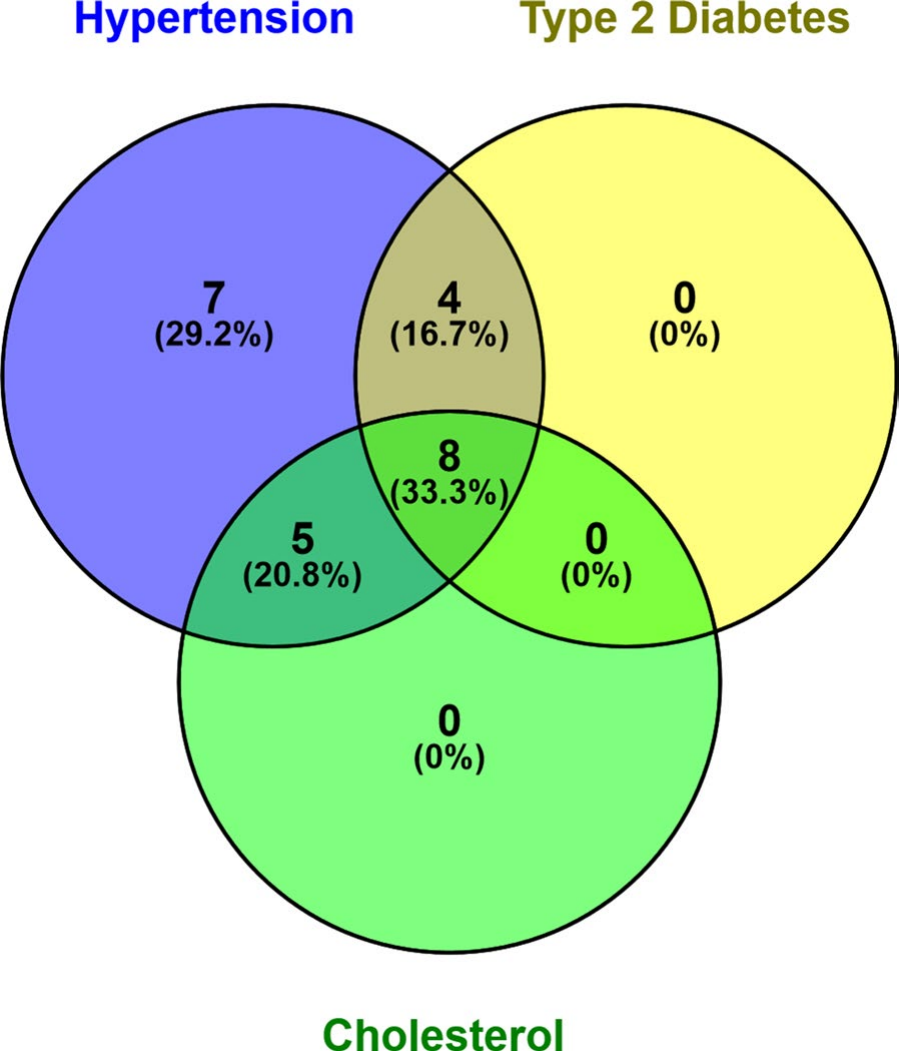
Participant Cardiovascular Burden. This figure illustrates the number and percentage of subjects in the present sample with one or more cardiovascular conditions (hypertension, type 2 diabetes, and high cholesterol)

Clinical and demographic information for the 24 participants is presented in Table 1. All participants had hypertension (mean duration = 9.9 years; sd = 6.4 years), and 12 also had type 2 diabetes (mean duration = 12.0 years; sd = 6.5 years). At the time of testing, 12 participants were also on a cholesterol-lowering medication (see Fig. 1). Regardless of medication status, however, 13 participants in the present sample had LDL-C levels above the recommended primary target of 2 mmol/L for those at moderate to high risk of cardiovascular disease (i.e., individuals with hypertension and/or type 2 diabetes; [37]). FRS ranged from 0.06 (considered low, < 10%) to 0.41 (considered high, > 20%). Mean white matter burden was relatively low (1.2 cc).

**Table 1:** Demographic and clinical information

	Mean (sd) *n* = 24
Age (years)	70.6 (4.4)
Sex (male/female)	12/12
Education (years)	15.8 (2.9)
Shipley vocabulary (T score)	59.6 (6.4)
WASI matrix reasoning (T score)	62.5 (8.6)
Body mass index (kg/m^2^)	26.1 (2.4)
Systolic blood pressure (mmHg)	131.6 (16.9)
Diastolic blood pressure (mmHg)	74.5 (9.5)
Fasting glucose (mmol/L)	6.2 (1.2)
HbA1c (%)	6.3 (.73)
Total cholesterol (mmol/L)	4.6 (1.3)
HDL-C (mmol/L)	1.7 (0.4)
LDL-C (mmol/L)	2.4 (1.0)
Framingham cardiovascular disease risk (10-year probability)	0.23 (0.11)
Volume—white matter hyperintensities (cc)	1.2 (1.7)
Total learning (/80)	66.8 (8.9)

*WASI* Wechsler Adult Scale of Intelligence, *HbA1c* hemoglobin A1C, *HDL-C* high-density lipoprotein cholesterol, *LDL-C* low-density lipoprotein cholesterol

### Cardiovascular burden, age, and task performance

There were no significant correlations between FRS and age, between FRS and task performance (TotLrn), or between age or education and task performance.

### Cardiovascular burden and brain structure

There was no association between normalized total gray matter volume and age, however there was a significant, negative correlation between normalized total gray matter volume and FRS, *r* (26) = − 0.41, *p* = 0.03, where individuals with higher FRS had lower gray matter volume. Within the medial temporal lobe a different pattern emerged; both left and right hippocampal volumes were negatively associated with age (left: *r* (26) = − 0.37, *p* = 0.05; right: *r* (26) = − 0.41, *p* = 0.03), but not with FRS.

### Cardiovascular burden, learning, and hippocampal encoding-related (PPI) connectivity

Before carrying out the group-level analysis designed specifically to identify brain regions showing associations among left and right hippocampal PPI connectivity, cardiovascular burden (i.e., FRS), and cognitive task performance, we used 3dttest++ (AFNI) to confirm a main effect of task. Indeed, at the group level there was a very strong main effect of task, with both right and left hippocampus showing highly significant and widespread encoding-related connectivity with a number of brain regions including medial prefrontal regions and other regions of the default mode network as well as primary visual and visual association cortices. To isolate the strongest regions of activation, the resultant voxelwise statistical maps were thresholded by setting the false discovery rate (FDR) correction for multiple comparisons at q ≤ 0.0001. There was a main effect of task on encoding-related connectivity between the left hippocampus and a large portion of both right and left lateral posterior parietal and occipital regions. On the right, peak activation centered in the lingual gyrus/middle occipital gyrus but extended down into the right inferior occipital gyrus, inferior and middle temporal gyrus, and fusiform gyrus, and up into the right superior occipital gyrus and precuneus. On the left, peak activation centered in the middle occipital gyrus/middle temporal gyrus but extended into the left lingual gyrus, superior occipital gyrus, precuneus, and superior parietal lobule. There was also a main effect of task on encoding-related connectivity between the left hippocampus and right anterior cingulate cortex, left medial frontal gyrus, left superior temporal gyrus, left cingulate, left fusiform gyrus, and right and left medial temporal poles. These results are summarized in Table 2.

**Table 2:** Regions showing a main effect of task with left hippocampal connectivity

Region	Cluster size (*number of voxels*)	Coordinates of peak voxel^a^ (*Talairach space*)	T-score at peak voxel
Right lateral posterior parietal and occipital regions	874	−23, 85, 5	14.08
Left lateral posterior parietal and occipital regions	716	31, 73, 17	12.53
Right anterior cingulate cortex	71	−5, −44, 5	10.73
Left medial frontal gyrus	21	7, −59, 17	9.61
Left medial frontal gyrus	12	13, −50, 8	9.05
Left superior temporal gyrus	50	43, 46, 14	9.68
Left cingulate	23	7, 28, 32	8.74
Left fusiform gyrus	19	34, 40, −13	11.06
Right medial temporal pole	29	−38, −23, −25	8.06
Left medial temporal pole	22	28, −17, −31	7.23

Voxelwise statistical maps were thresholded by setting the false discovery rate (FDR) correction for multiple comparisons at q < 0.0001 (T-score 5.185)

^a^RAI order

Similarly, there was a main effect of task on encoding-related connectivity between the right hippocampus and a large portion of both right and left lateral posterior parietal and occipital regions. On the right, peak activation centered in the right cuneus/right middle occipital gyrus but extended into the right superior occipital gyrus, middle temporal gyrus, and fusiform gyrus. On the left, peak activation centered in the middle occipital gyrus but extended up into the left superior occipital gyrus, superior temporal gyrus, precuneus, and superior parietal lobule, and down into the left lingual gyrus, fusiform gyrus, and parahippocampal gyrus. There was also a main effect of task on encoding-related connectivity between the right hippocampus and right medial frontal gyrus/right anterior cingulate cortex extending into left medial frontal gyrus/left anterior cingulate, and between right hippocampus and right superior parietal lobe, right insula, left posterior cingulate, and right fusiform gyrus. These results are summarized in Table 3.

**Table 3:** Regions showing a main effect of task with right hippocampal connectivity

Region	Cluster size (*number of voxels*)	Coordinates of peak voxel^a^ (*Talairach space*)	T-score at peak voxel
Right lateral posterior parietal and occipital regions	527	−23, 82, 8	9.38
Left lateral posterior parietal and occipital regions	908	34, 88, 5	9.35
Right and left medial frontal gyrus/anterior cingulate cortex	336	−17, −32, 26	9.66
Right superior parietal lobe	121	−26, 49, 41	8.49
Right insula	96	−29, −29, 11	7.85
Left posterior cingulate	59	1, 28, 23	8.74
Right fusiform gyrus	29	−29, 40, −13	7.33

Voxelwise statistical maps were thresholded by setting the false discovery rate (FDR) correction for multiple comparisons at q = 0.0001 (T-score 5.761)

^a^RAI order

**Fig. 2:**
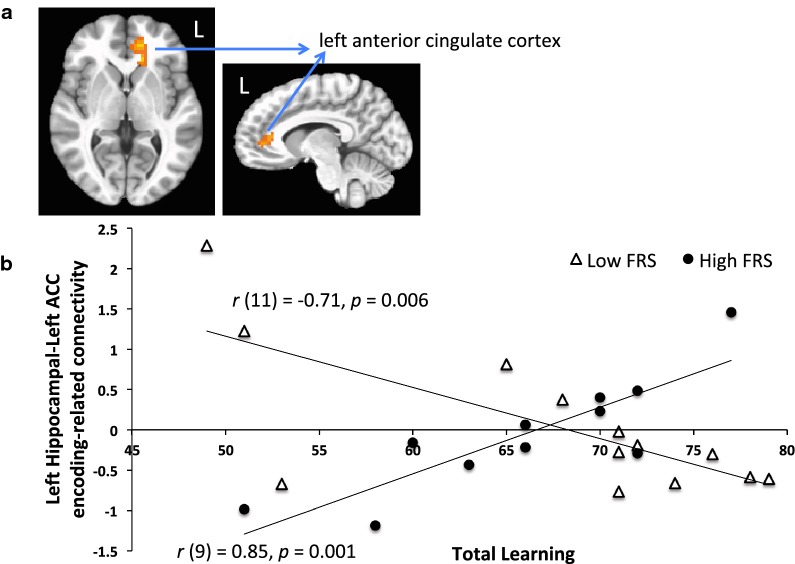
**a** The left hippocampal PPI analysis revealed that connectivity with the left anterior cingulate cortex during learning was significant and affected by FRS status. **b** To visualize the interaction, the sample is divided into a low- versus high-risk group according to FRS (HIGH: 10-year risk > 20%, average risk = 33%; LOW: 10-year risk < 20%, average risk = 14%). Encoding-related (PPI) connectivity between the left hippocampus and left anterior cingulate cortex regions is plotted against task performance (total words learned)

Following up with our analysis of interest, we found a significant interaction of FRS and TotLrn on PPI connectivity between the left hippocampus and primarily left midline prefrontal regions, comprising the left ventral anterior cingulate cortex and medial prefrontal cortex (114 voxels, *p* < 0.01; Fig. 2a). Specifically, in those with lower FRS, lower PPI connectivity between these regions was associated with better task performance, *r* (11) = − 0.71, *p *= 0.006. In those with higher FRS, stronger PPI connectivity between these regions was associated with better task performance (*r* (9) = 0.85, *p *= 0.001). To visualize the interaction (Fig. 2b), we divided the sample into a low/intermediate-versus high-risk group according to FRS. The low/intermediate-risk group (10-year risk < 20%) had an average score of 14%. The high-risk group (10-year risk > 20%) had an average score of 33%. As expected, the difference between groups was significant, *t*(22) = 7.78, *p *< 0.001.

There were no significant associations between left hippocampal PPI connectivity and left hippocampal volume or FRS. In addition, there were no significant interactions of FRS and TotLrn on right hippocampal PPI connectivity with any brain region, and there were no significant associations between right hippocampal PPI connectivity and hippocampal volume or FRS.

### Cerebrovascular reactivity and hippocampal encoding-related (PPI) connectivity

The five participants in the final sample who were non-compliant with the breath-hold paradigm were comparable to the compliant participants on most clinical and demographic measures, except they had lower scores on a test of non-verbal reasoning [mean (sd) = 53.8 (9.4) versus 64.8 (6.9), *F* (1, 22) = 8.8, *p *= 0.01] and learned fewer words on the list-learning task [mean (sd) = 59.8 (9.0) versus 68.6 (8.1), *F* (1, 22) = 4.5, *p *= 0.05]. In addition, systolic blood pressure in the non-compliant individuals was lower than in the compliant individuals [mean (sd) = 117.6 (15.4) versus 135.3 (15.7), *F* (1, 22) = 5.1, *p *= 0.03], as was FRS [mean (sd) = 0.14 (0.07) versus 0.26 (0.11), *F* (1, 22) = 4.6, *p *= 0.04].

In the subsample of participants with valid breath-hold data, there were positive associations between cerebrovascular reactivity and left hippocampal PPI connectivity with the right lingual gyrus (51 voxels, *p *< 0.01). There were also positive associations between cerebrovascular reactivity and right hippocampal PPI connectivity with a similar region of the right lingual gyrus, but also extending into the right inferior and middle occipital and fusiform gyri (274 voxels, *p* < 0.05).

## Discussion

Findings from the current study show that amongst older adults with cardiovascular conditions, those with high Framingham risk scores showed an altered relationship between left hippocampal-medial prefrontal coupling and task performance compared to those with low Framingham risk scores, which could reflect a compensatory mechanism in support of memory function. In addition, higher Framingham risk was associated with lower brain volumes. These results were obtained despite the relative homogeneity of our sample, restricted to cognitively-intact and well-educated older adults who all had some degree of cardiovascular burden (i.e., hypertension, type 2 diabetes, high cholesterol), but who were otherwise healthy, without any significant white matter pathology or vascular complications.

More specifically, the results showed a differential pattern of association based on cardiovascular burden, between task performance and encoding-related connectivity between the left hippocampus and a medial prefrontal region, where those with low/intermediate cardiovascular risk scores showed a pattern of weaker connectivity associated with better task performance, and those with higher cardiovascular burden showed the opposite pattern; stronger connectivity between regions was associated with better task performance.

The midline prefrontal region included left ventral anterior cingulate and medial prefrontal cortex, regions that, along with the precuneus and posterior cingulate cortex, comprise key hubs of the default mode network [38]. In the context of memory, activation studies have shown that default mode regions, including midline prefrontal regions and the precuneus, increase their activity during retrieval but deactivate during encoding [39]. On one hand, it has been suggested this reflects an inward orientation of attention during retrieval, and an outward orientation of attention during encoding (i.e., towards the to-be-learned stimuli; [40]) which is consistent with the observation that the default mode network is most active during rest states and periods of internally-directed attention and thought [41]. Another account, based on the observation that activity in the hippocampus tends to increase with *both* successful retrieval and successful encoding, suggests that the default mode network is coupled with the hippocampus during retrieval, but decoupled during encoding [42]. Consistent with this latter account, the present study found that in those with lower cardiovascular risk scores, weaker encoding-related coupling between the hippocampus and medial prefrontal regions was associated with better task performance. The finding of altered hippocampal coupling with medial prefrontal regions in the higher Framingham risk group suggests that these regions may be the first to show early signs of dysfunction associated with metabolic and vascular burden. This is consistent with work showing that default mode regions are particularly vulnerable to vascular and metabolic disruption; for instance, elevated Framingham Risk has been associated with reduced glucose metabolism and resting cerebral blood flow declines in midline prefrontal regions [43, 44], and the presence of multiple cardiovascular conditions has been shown to affect cerebrovascular reactivity across default mode network regions [45]. These results suggest that changes in the pattern of coupling between left hippocampal and default mode regions during memory task performance may be an early indicator of dysfunction within the episodic memory system associated with vascular and metabolic disruption.

Given the direction of association with task performance in those with higher cardiovascular risk scores, stronger coupling between the hippocampus and default mode regions appears to reflect engagement of compensatory mechanisms to support learning and sustain performance. This is consistent with other work; for example, cognitively-intact older adults without amyloid burden showed increases relative to young adults in encoding-related connectivity between medial temporal regions and the bilateral anterior cingulate, linked to successful task performance [46]. With respect to the present sample, the lack of direct association between cardiovascular burden and task performance suggests that the observed connectivity changes are an early sign of inefficiency within memory networks, before compensatory processes break-down and behavioral changes become evident.

Further, these connectivity changes were present in brain regions where cerebrovascular reactivity and connectivity were unrelated, which suggests they are occurring independently of cerebrovascular health. Whether the observed functional changes reflect a higher burden of amyloid pathology in individuals with higher cardiovascular burden, as might be predicted from the literature (e.g., [47]), could be a focus of future work. Indeed, accumulation of amyloid and tau pathology, even in the absence of cognitive impairment, can occur in normally aging adults (e.g., [48]), and early amyloid deposition tends to occur preferentially in default mode network regions [19]. Ultimately, the emergence of amnestic mild cognitive impairment and progression to Alzheimer disease depends on the accumulating burden of pathology, and on the ability of brain networks to adapt to this disruption. In the presence of cardiovascular conditions, this normal, age-related accumulation of amyloid and tau burden is exacerbated, resulting in a pattern of “accelerated aging” that increases risk of mild cognitive impairment and dementia in these individuals (e.g., [49]). This is consistent with population-based studies that show an earlier and faster progression of cognitive decline associated with cardiovascular burden (e.g., [50]), and with findings from the present study that offer evidence for structural volume loss and early disruptions in memory function and connectivity associated with higher cardiovascular risk in cognitively-normal older adults.

In addition, it will be important to examine how coupling between hippocampal and default mode regions changes when metabolic dysregulation is more pronounced and vascular burden is higher; in the present sample, the participants’ conditions were under very good control. How coupling between hippocampal and midline prefrontal regions changes when behavioral deficits become evident will also need to be addressed.

Lastly, there are some limitations to the present analysis. First, there was no explicit control condition for the word-list learning task nor was there a healthy control group, which does limit interpretation of the findings. In addition, the region of interest identified by the group analysis on left hippocampal PPI connectivity (left anterior cingulate cortex) was in close proximity to regions of the brain typically affected by susceptibility artefacts during scanning. As described above, we addressed this in our analysis by masking regions of the brain with valid EPI signal and including only those voxels in the analysis; however, the proximity to affected regions may have had some degree of impact on the findings.

## Conclusions

Taken together, findings from the current study show that amongst older adults with cardiovascular conditions, those with higher Framingham risk scores had lower brain volumes and an altered relationship between left hippocampal-medial prefrontal coupling and task performance compared to those with low Framingham risk scores, which appears to be a compensatory mechanism that supports memory function. Although not assessed directly in the present study, these changes appear to parallel those associated with normal aging and progression to amnestic mild cognitive impairment and Alzheimer disease, which suggests that the underlying mechanisms may be similar, and that older adults with elevated cardiovascular risk are vulnerable to early Alzheimer disease-like dysfunction within the episodic memory system.

## Data Availability

The data supporting these results is stored at the Baycrest Centre for Geriatric Care in Toronto, Canada. The datasets analyzed are available from the corresponding author on reasonable request.

## Abbreviations

AFNI: Analysis of Functional NeuroImages
BOLD: blood oxygenation level dependent
FLAIR: fluid attenuation inversion recovery
fMRI: functional magnetic resonance imaging
FRS: Framingham risk score
FSL: FMRIB Software Library
HbA1c: hemoglobin A1C
HDL-C: high-density lipoprotein cholesterol
LDL-C: low-density lipoprotein cholesterol
PPI: psychophysiological interaction
TE: echo time
TI: inversion time
TR: repetition time
TotLrn: total number of words learned across all 5 trials of the word-list learning task
WASI: Wechsler Adult Scale of Intelligence
